# Elevated Levels of Urinary Markers of Oxidative DNA and RNA Damage in Type 2 Diabetes with Complications

**DOI:** 10.1155/2016/4323198

**Published:** 2015-12-07

**Authors:** Xinle Liu, Wei Gan, Yuangao Zou, Bin Yang, Zhenzhen Su, Jin Deng, Lanlan Wang, Jianping Cai

**Affiliations:** ^1^Department of Lab Medicine, West China Hospital, Sichuan University, Chengdu, Sichuan 610041, China; ^2^The Key Laboratory of Geriatrics, Beijing Hospital and Beijing Institute of Geriatrics, Ministry of Health, Beijing 100730, China

## Abstract

The mechanisms underlying progression of type 2 diabetes are complex and varied. Recent studies indicated that oxidative stress provided a new sight. To further assess the relationship between nucleic acid oxidation and complications in patients with type 2 diabetes and explore its possible molecular mechanisms, we studied 1316 subjects, including 633 type 2 diabetes patients and 683 age- and sex-matched healthy controls. Urinary levels of DNA oxidation marker 8-oxo-7,8-dihydro-2′-deoxyguanosine (8-oxodGuo) and RNA oxidation marker 8-oxo-7,8-dihydroguanosine (8-oxoGuo) were measured by ultraperformance liquid chromatography and mass spectrometry (UPLC-MS/MS). Serum glucose, HbA1c, total cholesterol, HDL cholesterol, LDL cholesterol, and triglycerides (TG) were also determined. The results showed significantly elevated levels of both the urinary 8-oxodGuo and 8-oxoGuo in diabetes patients with/without complications compared with age-matched healthy control subjects (*p* = 0.02 and *p* < 0.001, resp.). Patients with complications, especially macrovascular complications, exhibited higher levels of 8-oxoGuo than those without complications, while there was no difference in the concentrations of serum glucose and lipids. The finding indicates the role for oxidative damage to DNA and RNA, as a molecular mechanism contributing to the progression of type 2 diabetes. Elevated levels of 8-oxoGuo may be a risk factor for type 2 diabetes complications, especially in diabetic macrovascular complications.

## 1. Introduction

Type 2 diabetes is a chronic disease characterized by hyperglycemia in the context of insulin resistance and relative lack of insulin. About 382 million people were living with diabetes in 2013 according to the International Diabetes Federation, and by 2035 the number will be almost 600 million [1]. As the prevalence of diabetes has risen to epidemic proportions worldwide, the complications of diabetes have now become one of the most challenging health problems [2]. Thus, new biomarkers that can be used for risk stratification and therapy control as an alternative to current methods are needed.

It has been reported that the occurrence of diabetic complications increased with poor glycaemic control. However, we found that diabetic complications also occurred with better glycaemic control. So there may be other mechanisms for the progression of diabetic complications. Some previous researches on both epidemiology and mechanics suggested that oxidative stress played a pivotal role in the pathogenesis and progression of type 2 diabetes complications [3–7]. And the increased oxidative stress in patients with type 2 diabetes can be caused by hyperglycemia, inflammation, or dyslipidemia [8]. Historically, DNA was the focus of nucleic acid oxidation studies [9, 10]. However, RNA also comprised an incredibly diverse group of nucleic acids and played vital functions in cellular function and protein synthesis [11]. 8-Oxo-7,8-dihydro-2′-deoxyguanosine (8-oxodGuo) and 8-oxo-7,8-dihydroguanosine (8-oxoGuo) are two main nucleic acid oxidative adducts derived from DNA and RNA, respectively. Many previous studies have shown that levels of urinary 8-oxodGuo were elevated in diabetes patients with complications; however, the evidence on the association between urinary 8-oxoGuo and diabetes complications was relatively little [12, 13].

In this study, we analyzed the levels of urinary 8-oxodGuo and 8-oxoGuo, serum glucose, and lipids in patients with diabetes and healthy controls. In addition, we investigated the association between 8-oxoGuo and different complications of type 2 diabetes.

## 2. Materials and Methods

### 2.1. Subjects

The study group consisted of 1,316 subjects, including 633 type 2 diabetes patients and 683 age- and sex-matched healthy controls. All the subjects were recruited from West China Hospital of Sichuan University from January 2014 to October 2014. In the 633 type 2 diabetes patients, 267 patients had complications such as retinopathy, nephropathy, neuropathy, and other vascular complications; the other 366 patients had no complication. All the patients were diagnosed according to the World Health Organization (WHO) diagnostic criteria for type 2 diabetes [14]. Members of the control group, who did not have any personal or family history of either diabetes or dyslipidemia and with normal thyroid, hepatic, and renal functions, were selected from the Health Examination Center of West China Hospital. The study was approved by the Institutional Ethics Committee of West China Hospital of Sichuan University and complied with Declaration of Helsinki. Written informed consent was obtained prior to enrollment from all subjects.

### 2.2. Measurement of Biochemical Parameters

Blood samples were collected at the fasting state from each subject. Total cholesterol, HDL cholesterol, LDL cholesterol, and triglycerides (TG) were analyzed enzymatically in serum (Modular P800, Roche Diagnostics GmbH, Germany). Glucose was enzymatically determined by using the hexokinase method (Modular P800, Roche Diagnostics GmbH, Germany). HbA1c was determined by a method based on high-performance liquid chromatography (HPLC) which was approved by National Glycohemoglobin Standardization Program (NGSP) (HLC-723 G8, Tosoh Corporation, Japan) in whole blood. Urinary creatinine was analyzed by the Jaffe reaction (Modular P800, Roche Diagnostics GmbH, Germany).

### 2.3. Measurement of Urinary 8-oxodGuo and 8-oxoGuo

Freshly voided urine samples were obtained from each subject and centrifuged; after that, the supernatants of 1.0 mL were transferred to Eppendorf tubes and stored at −80°C until they were analyzed. The frozen samples were thawed, mixed, and heated to 37°C for 5 min and then centrifuged at 10,000 g for 5 min. The supernatant was used for the analysis. Samples from patients and healthy control subjects were plated on the assay plate in a randomly assigned sequence. [^15^N_2_^13^C_1_]8-oxoGuo, the stable isotope labeled 8-oxoGuo, was customized from Toronto Research Chemicals (Canada). 8-oxodGuo (>98% purity) was purchased from Sigma-Aldrich (USA). 8-oxoGuo (>98% purity) was purchased from ALEXIS Biochemicals (USA). The urinary content of the oxidized nucleosides 8-oxodGuo and 8-oxoGuo was quantified using a modified ultraperformance liquid chromatography and mass spectrometry (UPLC-MS/MS) assay established by our laboratory. Briefly, the chromatographic separation was performed on an Acquity UPLC system (Waters Corporation, Milford, USA) using an Acquity UPLC BEH C18 column (1.7 *μ*m, 2.1 × 50 mm; Waters Corp.) and a VanGuard precolumn (1.7 *μ*m, 2.1 × 5 mm; Waters Corp.) with a column temperature of 25°C. A waters ACQUITY UPLC equipped with an Xevo TQ-S triple quadrupole mass spectrometer was used for measurement.

The mobile phase contained 0.1% (v/v) formic acid (A) and 100% methanol (B); gradient elution was applied to obtain the best peak shape (see Supplementary Table  1 in Supplementary Material available online at http://dx.doi.org/10.1155/2016/4323198). Electrospray ionization was performed in the positive ion mode. The multiple reaction monitoring (MRM) mode was applied during quantification. The desolvation temperature was set at 500°C. Capillary and cone voltages were set at 2.5 KV and 20 V. Quantification was based on the signal peak area from transitions *m*/*z* 283.9 → 167.9 (8-oxodGuo) and 299.9 → 167.9 (8-oxoGuo) related to the peak area of the [^15^N_2_^13^C_1_]8-oxoGuo *m*/*z* 302.9 → 170.9. 8-oxodGuo and 8-oxoGuo were eluted at 1.63 and 1.32 minutes, respectively. Considering the variability among the urinary volumes and the significant differences in the renal glomerular function, urinary levels of 8-oxodGuo and 8-oxoGuo were normalized to the urinary creatinine concentration. Laboratory personnel performing the analysis were blinded to the category of participants and the clinical state of patients with type 2 diabetes.

### 2.4. Statistical Analysis

The clinical characteristics of the participants were expressed as mean ± SD for normally distributed variables and were compared between groups by independent *t*-test. The* chi-square* test was used to determine the significance of the differences in the distribution of categorical data. Pearson's correlation analysis was performed to examine the relationships between nucleotide acid oxidation markers and factors of interest. Ordinal regression analysis was further used to determine the relationships between nucleotide acid oxidation markers and the variables. For all parametric tests, statistical analyses were performed using SPSS 21.0 software (IBM Corporation, New York, NY, USA) and were plotted with GraphPad Prism 5.0 software (GraphPad Software Inc., La Jolla, CA, USA). Additional information on statistical methods is available in the Supplementary Material. A two-sided *p* value < 0.05 was deemed statistically significant.

## 3. Results

### 3.1. Demographic and Clinical Characteristics

Demographic and clinical characteristics of the study subjects are described in Table 1. All of the study participants were Chinese and the patients received specialized treatment at the Endocrinology Department of West China Hospital. There were no significant differences in age and gender between healthy controls and diabetes patients. Glucose levels were significantly higher in patients with type 2 diabetes in contrast with the healthy controls (*p* < 0.001). There was no significant difference between diabetes patients with complications and those without complications in HbA1c (7.76 ± 1.93 versus 8.00 ± 4.27%, *p* = 0.129). Blood lipid levels such as triglyceride, total cholesterol, HDL, and LDL cholesterol also had no significant differences between diabetes patients with complications and those without complications.

### 3.2. Higher Levels of 8-oxodGuo and 8-oxoGuo in Diabetes Patients Than in Healthy Controls

**Figure 1:**
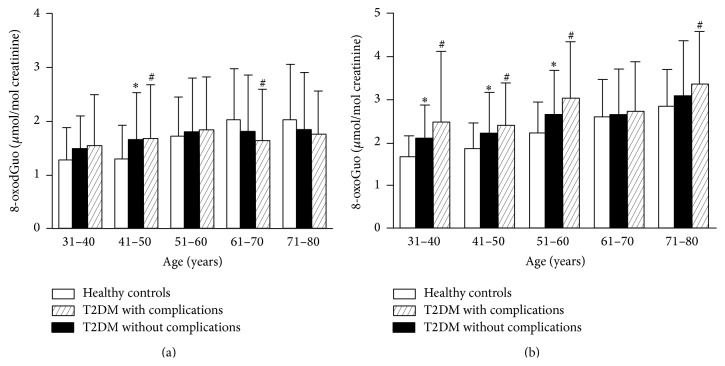
Urinary levels of 8-oxodGuo or 8-oxoGuo with age in healthy controls and patients. (a) Urinary 8-oxodGuo (*μ*mol/mol creatinine) levels in healthy controls and patients with type 2 diabetes. (b) Urinary 8-oxoGuo (*μ*mol/mol creatinine) levels in healthy controls and patients with type 2 diabetes. Columns represent mean ± SD. ^*∗*^ *p* < 0.05 (statistical differences between healthy controls and diabetes patients without complications by Student's *t*-test). ^#^ *p* < 0.05 (statistical differences between T2DM with complications and diabetes patients without complications by Student's *t*-test). T2DM: type 2 diabetes mellitus.

An UPLC-MS/MS chromatogram of [^15^N_2_^13^C_1_]8-oxoGuo, 8-oxoGuo, and 8-oxodGuo is shown in Supplementary Figure  1A together with a chromatogram of a urine sample (Supplementary Figure  1B). The urinary levels of 8-oxodGuo and 8-oxoGuo in healthy controls and diabetes patients are showed in Table 2. We noted that age played an important role in both the DNA marker 8-oxodGuo and the RNA marker 8-oxoGuo. Therefore, we subdivided all the participants into different groups with each group every 10 years old and further analyzed them in different age groups (Table 2 and Figure 1). In healthy controls, both 8-oxodGuo and 8-oxoGuo increased with age. The correlation coefficient of urinary 8-oxoGuo with age was 0.50, which indicated a stronger degree of linear relationship than that of 8-oxodGuo with age (*r* = 0.35; *p* < 0.001; *n* = 683) (Supplementary Figure  2).

Correlations between nucleic acid oxidation markers and variables of interest are presented in Table 3. In order to assess the effects of different variables on 8-oxodGuo and 8-oxoGuo, we further performed ordinal regression analysis (Supplementary Table  2). The results also showed that age played an important part in nucleic acid oxidation of both 8-oxodGuo and 8-oxoGuo. Moreover, we compared the correlations between oxidative stress markers and HbA1c in adults with type 2 diabetes. There was a moderate correlation between 8-oxoGuo and HbA1c (*r* = 0.16; *p* < 0.001) (Supplementary Figure  3B). However, the correlation between 8-oxodGuo and HbA1c was not significant (*r* = 0.07; *p* > 0.05) (Supplementary Figure  3A). In addition, the levels of urinary 8-oxodGuo and 8-oxoGuo were significantly correlated (*r* = 0.642; *p* < 0.001; *n* = 1,316) (Supplementary Figure  4).

In all the participants, the levels of both 8-oxodGuo and 8-oxoGuo were significantly higher in diabetes with/without complications group compared with the age-matched control group (*p* = 0.02 and *p* < 0.001, resp.). Furthermore, we compared the changes of 8-oxodGuo and 8-oxoGuo in the patients with complications and those without complications. For 8-oxodGuo, it was slightly higher in diabetes with complications compared to those without complications with age between 31 and 60 years (Figure 1(a)). For 8-oxoGuo, there was a rather clear and significant increasing trend in diabetes with/without complications compared to age-matched healthy controls (Figure 1(b)). This increasing trend was seen in all the different age sets. And most pronounced difference was seen between diabetes patients with complications and those without complications (3.01 ± 1.32 versus 2.64 ± 1.04 *μ*mol/mol creatinine, *p* < 0.05) with age between 51 and 60 years (Table 2). And also we found that the levels of urinary 8-oxoGuo were obviously higher than 8-oxodGuo in the same age group of participants.

### 3.3. Levels of 8-oxoGuo in Diabetes Patients with Different Complications

**Figure 2:**
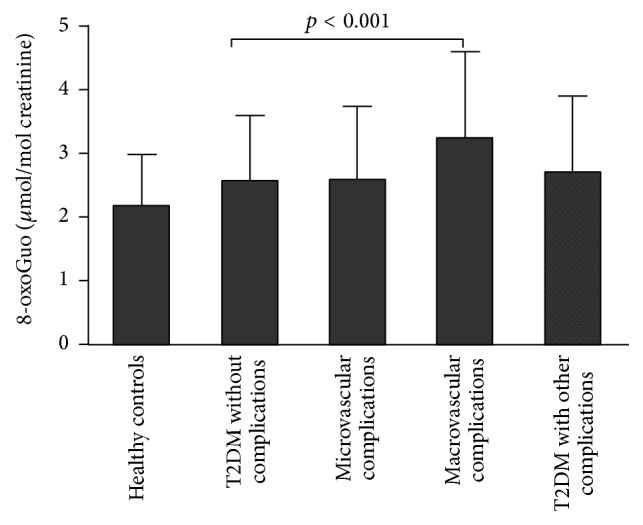
Urinary levels of 8-oxoGuo in diabetes patients with different complications. *p* < 0.01 (statistical differences between patients with macrovascular complications and patients without complications by Student's *t*-test).

There are many different types in diabetic complications, for example, microvascular complications and macrovascular complications. Considering that 8-oxoGuo had a more obvious increasing trend in diabetes complications than 8-oxodGuo, we analyzed the change of 8-oxoGuo levels in all the participants in Figure 2. Apparently, there was a significant difference in 8-oxoGuo between diabetes with macrovascular complications and those without complications (3.21 ± 1.36 versus 2.53 ± 1.05 *μ*mol/mol creatinine, *p* < 0.001). And patients with diabetic nephropathy had higher levels of 8-oxoGuo than those without complications, but not significant.

## 4. Discussion

With the increase of the diabetes incidence rate, more studies are needed to do research on its pathogenic mechanism and find the relevant therapies. Although the mechanisms underlying development of the disease are complex and varied, oxidative stress provides a new sight. We noted that many variables such as nucleic acid oxidation, serum glucose, and lipids had significant differences between diabetes patients and healthy controls, which was in accordance with some previous studies [15–17]. However, there were only 8-oxoGuo, but not serum glucose, and lipids that had significant differences between diabetes patients with complications and those without complications. We also found that age had a significant effect on both 8-oxodGuo and 8-oxoGuo in the study subjects. This confirmed our previous research [18, 19] and also the review by Jacob et al. [20].

In combination with the previously published results, all these results suggest that urinary 8-oxodGuo and 8-oxoGuo are potentially useful biomarkers for evaluating the severity of complications in patients with type 2 diabetes. Compared with DNA, RNA is more prone to oxidative damage because of its widespread distribution in close proximity to sites of reactive oxygen species generation, its single stranded nature, unidentified active repair mechanism, and the lesser association with protecting proteins [21]. However, the focus on nucleic acid oxidation research has been centered on DNA [22, 23]. We found that there was a clear and significant trend for increased risk in 8-oxoGuo between diabetes patients with complications compared to those without complications. However, the trend for 8-oxodGuo is moderate, which was consistent with previous studies [22, 24–27]. Further studies are needed to establish the RNA oxidation.

Some previous studies indicated that increased oxidative stress might have a primary role in the pathogenesis of diabetic complications, especially patients with nephropathy and vascular complications [22, 23, 28–31]. We also found that there was a significant difference in 8-oxoGuo between patients with macrovascular complications and those without complications. Broedbaek et al. [12] showed that urinary excretion of the RNA oxidation marker 8-oxoGuo independently predicted all-cause and diabetes-related mortality, whereas the DNA oxidation marker 8-oxodGuo did not. Therefore, urinary 8-oxoGuo could be a useful biomarker for diabetes with complications, especially in macrovascular complications.

It has been well known that cells have protective mechanisms against oxidative damage. However, oxidative stress occurs when the reactive oxygen species (ROS) level overwhelms defensive mechanisms, causing a cellular imbalance between pro- and antioxidant factors. Elevated levels of oxidation markers such as 8-oxodGuo and 8-oxoGuo probably lead to strand breaks and to oxidative base modifications; and multiple signaling pathways may also contribute to the adverse effects of glucotoxicity on cellular functions [8, 11]. Therefore, higher levels of 8-oxodGuo and 8-oxoGuo indicate diabetic complications. In this study, the findings not only provide further insights into the pathophysiological mechanisms responsible for the progression of complications in diabetes but also indicate that 8-oxoGuo has more advantages than 8-oxodGuo in clinical applications.

The main strengths of our study are as follows. First, our study employed UPLC-MS/MS for the measurement of DNA and RNA oxidation, a method that was more specific and reliable than the enzyme-linked immunosorbent assay (ELISA) in urinary samples [32–34]. Next, we investigated the variation between the two measurements of 8-oxodGuo and 8-oxoGuo one week apart in healthy individuals and found a variability of less than 10% in the preliminary experiment, similarly reported by Barregard et al. [33]. In addition, we tested 8-oxodGuo and 8-oxoGuo in the urine rather than in blood, which was mainly explained by the following three reasons. Firstly, blood levels of 8-oxodGuo and 8-oxoGuo were mainly determined by the kidney function and when different individuals were compared, they were thus unlikely to provide specific information about oxidative stress [24]. Secondly, urine contained a higher level of oxidized guanosine than blood, thus providing a higher accuracy. Thirdly, the sample of urine was obtained more easily and noninvasively than blood.

There are also some limitations in this study. First, consumption of medicines in diabetes patients was little known. Some studies reported that interventions such as antioxidants could decrease oxidative stress [35–37]. Second, glucose variability, which has been shown to exhibit a more specific triggering effect on oxidative stress than chronic sustained hyperglycemia [38–40], was not assessed in the study.

In summary, the present study demonstrated significantly higher levels of oxidatively generated damage to DNA and RNA in patients with type 2 diabetes compared with healthy control subjects. We suggest the role for 8-oxodGuo and 8-oxoGuo as a molecular mechanism contributing to the complications of type 2 diabetes. Nucleic acid oxidation, especially RNA oxidation, is a possible pathogenesis of type 2 diabetes complications. Further studies should be initiated to evaluate the potential clinical applications of 8-oxoGuo as a biomarker in diabetes that could be used for risk stratification, progressive course, selection of appropriate therapeutic intervention, and the monitoring of response to therapy.

## Supplementary Material

Supplementary material includes two tables and four figures. Supplementary Table 1 shows HPLC conditions for analytes. Supplementary Table 2 shows the effects of variables on urinary 8-oxodGuo and 8-oxoGuo by ordinal regression. Supplementary Figure 1 shows UPLC-MS/MS chromatograms. (A) Standard of CN-8-oxoG, 8-oxoGuo and 8-oxodGuo. (B) Human urine sample. CN-8-oxoG: the internal standard for 8-oxoGuo. Supplementary Figure 2 shows the correlation of nucleic acid oxidation with age in healthy controls. (A) Correlation of urinary 8-oxodGuo (µmol/mol creatinine) levels with age in healthy controls (r = 0.35, p < 0.001, n = 683). (B) Correlation of urinary 8-oxoGuo (µmol/mol creatinine) levels with age in healthy controls (r = 0.50, p < 0.001, n = 683). Supplementary Figure 3 shows the correlations between urinary biomarkers and HbA1c in patients with type 2 diabetes. (A) Between 8-oxodGuo and HbA1c. (B) Between 8-oxoGuo and HbA1c. Supplementary Figure 4 shows the correlation of urinary 8-oxodGuo with 8-oxoGuo.

## Figures and Tables

**Table 1:** Demographic and clinical characteristics of the study participants.

Variables	Healthy controls (*n* = 683)	T2DM			*p*
		All	Without complications	With complications	
		(*n* = 633)	(*n* = 366)	(*n* = 267)	
Age, years, mean ± SD^†^	57.1 ± 12.6	57.2 ± 12.4	57.6 ± 11.7	58.8 ± 11.6	0.12^*∗*^/0.176^*∗∗*^
Gender (male/female)^‡^	362/321	364/269	217/149	147/120	0.11^*∗*^/0.288^*∗∗*^
HbA1c (%)	—	7.90 ± 3.51	8.00 ± 4.27	7.76 ± 1.93	0.129^*∗∗*^
Fasting blood glucose (mmol/L)^†^	5.44 ± 0.75	8.68 ± 3.14	8.90 ± 3.22	8.38 ± 3.02	<0.001^*∗*^/0.03^*∗∗*^
Triglyceride (mmol/L)^†^	1.41 ± 0.94	1.64 ± 1.07	1.67 ± 1.08	1.59 ± 1.06	<0.001^*∗*^/0.47^*∗∗*^
Total cholesterol (mmol/L)^†^	4.95 ± 0.96	4.59 ± 1.10	4.60 ± 1.00	4.58 ± 1.23	<0.001^*∗*^/0.48^*∗∗*^
HDL cholesterol (mmol/L)^†^	1.58 ± 0.44	1.38 ± 0.44	1.35 ± 0.38	1.41 ± 0.50	<0.001^*∗*^/0.36^*∗∗*^
LDL cholesterol (mmol/L)^†^	2.91 ± 0.75	2.67 ± 0.94	2.67 ± 0.84	2.66 ± 1.07	<0.001^*∗*^/0.38^*∗∗*^

Data expressed as arithmetic mean ± standard deviation or *n*.

^†^Statistical differences between groups tested using unpaired Student's *t* test. ^‡^Statistical differences between groups tested using chi-square test. ^*∗*^Statistical differences between healthy controls and patients with T2DM. ^*∗∗*^Statistical differences between T2DM without complications and T2DM with complications.

**Table 2:** Urinary levels of 8-oxodGuo and 8-oxoGuo in the different age group.

	Age group					
	31–40 (years)	41–50 (years)	51–60 (years)	61–70 (years)	71–80 (years)	All {31–80 (years)}
8-oxodGuo (*μ*mol/mol creatinine)						
Healthy controls	1.27 ± 0.58	1.25 ± 0.56	1.73 ± 0.76	2.02 ± 0.95	2.02 ± 1.04	1.68 ± 0.87
Diabetes without complications	1.48 ± 0.62	1.65 ± 0.86	1.80 ± 1.01	1.80 ± 1.04	1.84 ± 1.07	1.75 ± 0.97
Diabetes with complications	1.53 ± 0.96	1.67 ± 1.00	1.84 ± 0.98	1.62 ± 0.97	1.75 ± 0.81	1.70 ± 0.95
8-oxoGuo (*μ*mol/mol creatinine)						
Healthy controls	1.65 ± 0.47	1.81 ± 0.55	2.22 ± 0.72	2.58 ± 0.88	2.82 ± 0.86	2.25 ± 0.84
Diabetes without complications	2.09 ± 0.78	2.19 ± 0.97	2.64 ± 1.04	2.63 ± 1.08	3.07 ± 1.29	2.55 ± 1.10
Diabetes with complications	2.45 ± 1.67	2.38 ± 1.01	3.01 ± 1.32	2.70 ± 1.17	3.34 ± 1.24	2.81 ± 1.27

**Table 3:** Pearson's correlation coefficients of nucleic acid oxidation with variables of interest.

Study subjects	All		Healthy controls		Type 2 diabetes	
	*r*	*p*	*r*	*p*	*r*	*p*
8-oxodGuo						
Age	0.208	<0.001	0.346	<0.001	0.062	0.120
Fasting blood glucose	0.019	0.509	0.074	0.063	0.004	0.919
Triglyceride	−0.042	0.140	−0.055	0.167	−0.038	0.365
Total cholesterol	−0.025	0.381	0.060	0.127	−0.092	0.028
HDL cholesterol	0.041	0.150	0.125	0.002	−0.029	0.494
LDL cholesterol	−0.057	0.048	0.015	0.709	−0.108	0.010
8-oxoGuo						
Age	0.350	<0.001	0.497	<0.001	0.239	<0.001
Fasting blood glucose	0.135	<0.001	0.095	0.016	0.019	0.649
Triglyceride	0.000	0.990	0.030	0.442	−0.059	0.159
Total cholesterol	−0.037	0.199	0.099	0.012	−0.074	0.078
HDL cholesterol	0.009	0.750	0.122	0.002	0.002	0.953
LDL cholesterol	−0.073	0.011	0.007	0.854	−0.081	0.055
