# Supplementary material for: Elevated Levels of Urinary Markers of Oxidative DNA and RNA Damage in Type 2 Diabetes with Complications
Source: Oxid Med Cell Longev. 2015 Dec 7;2016:4323198. doi: 10.1155/2016/4323198 (PMC4685146; doi:10.1155/2016/4323198)
Supplement: Supplementary file 1 — Supplementary material includes two tables and four figures. Supplementary Table 1 shows HPLC conditions for analytes. Supplementary Table 2 shows the effects of variables on urinary 8-oxodGuo and 8-oxoGuo by ordinal regression. Supplementary Figure 1 shows UPLC-MS/MS chromatograms. (A) Standard of CN-8-oxoG, 8-oxoGuo and 8-oxodGuo. (B) Human urine sample. CN-8-oxoG: the internal standard for 8-oxoGuo. Supplementary Figure 2 shows the correlation of nucleic acid oxidation with age in healthy controls. (A) Correlation of urinary 8-oxodGuo (µmol/mol creatinine) levels with age in healthy controls (r = 0.35, p < 0.001, n = 683). (B) Correlation of urinary 8-oxoGuo (µmol/mol creatinine) levels with age in healthy controls (r = 0.50, p < 0.001, n = 683). Supplementary Figure 3 shows the correlations between urinary biomarkers and HbA1c in patients with type 2 diabetes. (A) Between 8-oxodGuo and HbA1c. (B) Between 8-oxoGuo and HbA1c. Supplementary Figure 4 shows the correlation of urinary 8-oxodGuo with 8-oxoGuo. [file 4323198.f1.pdf]

Supplementary table 1 HPLC conditions for analytes

Analytes	Elute condition	Injection volume	Run time
8-oxodGuo and 8-oxoGuo in urine	0-1.8 min: 5% B, 0.3ml/min;<br>1.8-2.0 min: 20% B, 0.3ml/min;<br>2.0-3.0 min: 5% B, 0.3ml/min.	2 μl	3 min

Supplementary table 2 Effects of variables on urinary 8-oxodGuo and 8-oxoGuo by ordinal regression

Oxidation markers	Variables	OR (95% CI)	p
8-oxodGuo			
All	Age	1.03 (1.02-1.04)	<0.001
Healthy controls	Age	1.05 (1.04-1.06)	<0.001
8-oxoGuo			
All	Age	1.07 (1.06-1.08)	<0.001
	Glucose	1.12 (1.08-1.17)	<0.001
Healthy controls	Age	1.09 (1.07-1.10)	<0.001
Type 2 diabetes	Age	1.05 (1.03-1.06)	<0.001
	Glucose	1.07 (1.01-1.12)	0.013

OR, odds ratio; CI, confidence interval.

8-oxodGuo and 8-oxoGuo are categorized into four groups according to their quartiles respectively.

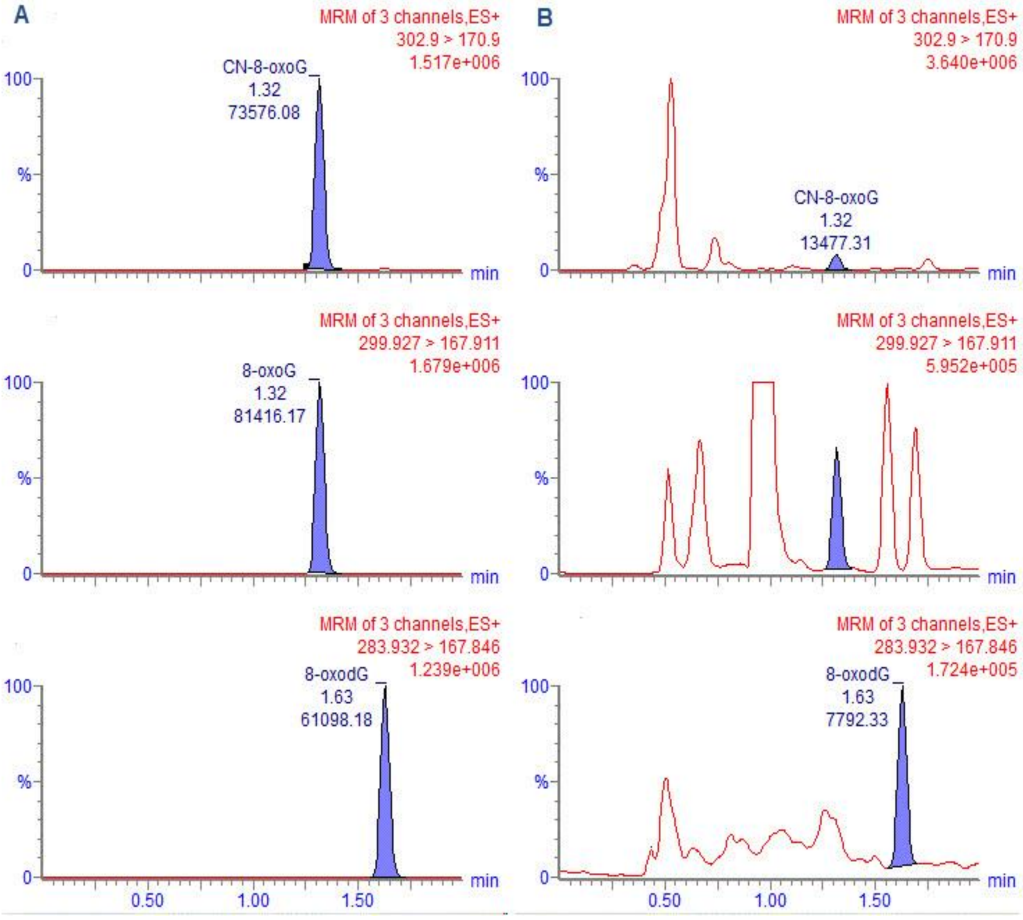

Supplementary figure 1 UPLC-MS/MS chromatograms. (A) Standard of CN-8-oxoG, 8-oxoGuo and 8-oxodGuo. (B) Human urine sample. CN-8-oxoG: $[^{15}N_2{}^{13}C_1]$8-oxoGuo, the internal standard for 8-oxoGuo. 8-oxodGuo and 8-oxoGuo were eluted at 1.63 and 1.32 minutes respectively.

A.

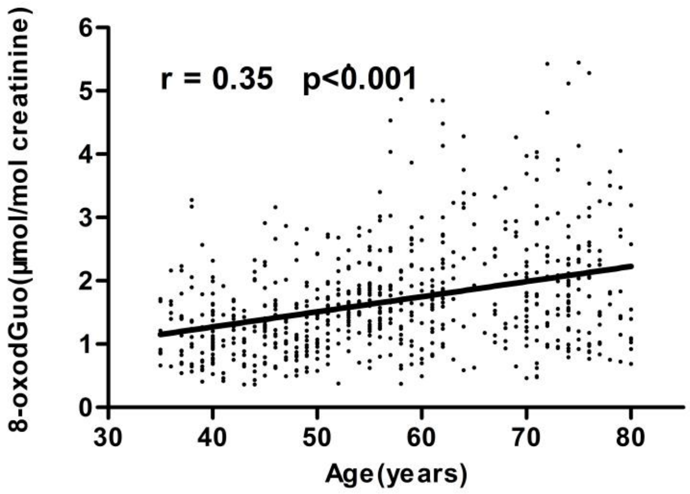

B.

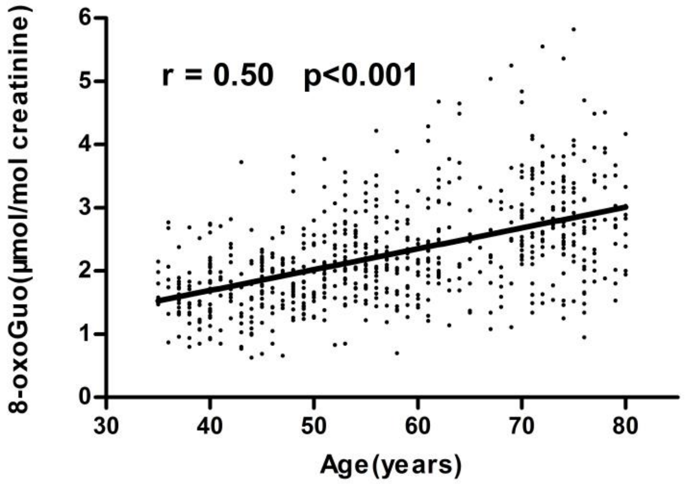

Supplementary figure 2 Correlation of nucleic acid oxidation with age in healthy controls. (A) Correlation of urinary 8-oxodGuo (μmol/mol creatinine) levels with age in healthy controls (r=0.35, p<0.001, n=683). (B) Correlation of urinary 8-oxoGuo (μmol/mol creatinine) levels with age in healthy controls (r=0.50, p<0.001, n=683).

A.

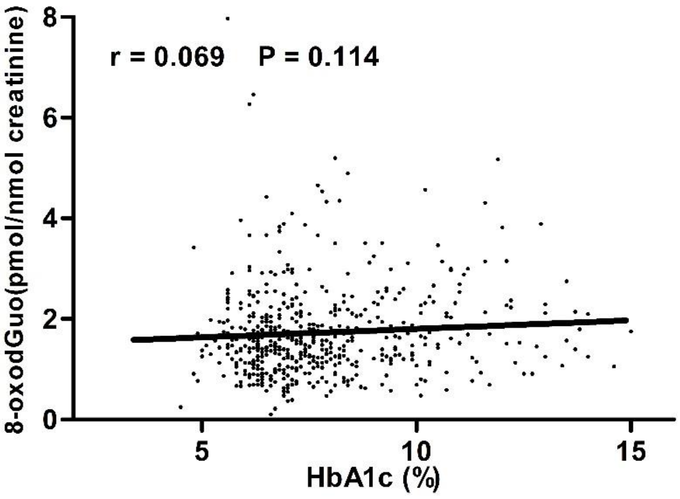

B.

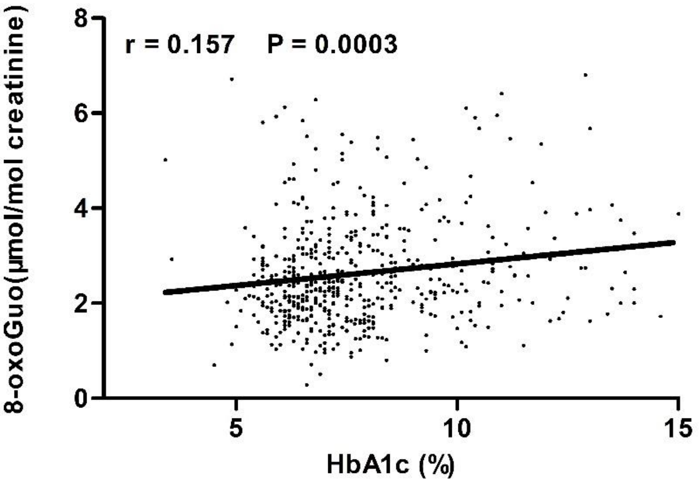

Supplementary figure 3 Correlations between urinary biomarkers and HbA1c in patients with type 2 diabetes. (A) Between 8-oxodGuo and HbA1c. (B) Between 8-oxoGuo and HbA1c.

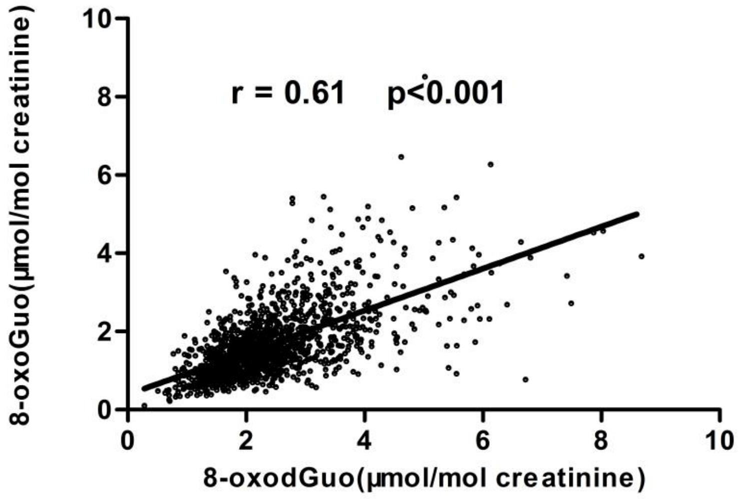

Supplementary figure 4 Correlation of urinary 8-oxodGuo with 8-oxoGuo.

Urinary levels of 8-oxodGuo and 8-oxoGuo in all the healthy controls and diabetes patients from 31 to 80 years ($r=0.61$, $p<0.001$, $n=1316$).
